# Characterizing the Coding Region Determinant-Binding Protein (CRD-BP)-Microphthalmia-associated Transcription Factor (MITF) mRNA interaction

**DOI:** 10.1371/journal.pone.0171196

**Published:** 2017-02-09

**Authors:** Gerrit van Rensburg, Sebastian Mackedenski, Chow H. Lee

**Affiliations:** Chemistry Program, University of Northern British Columbia, Prince George, British Columbia, Canada; Centre National de la Recherche Scientifique, FRANCE

## Abstract

Coding region determinant-binding protein (CRD-BP) binds to the 3’-UTR of microphthalmia-associated transcription factor (MITF) mRNA to prevent its targeted degradation by miR-340. Here, we aim to further understand the molecular interaction between CRD-BP and MITF RNA. Using point mutation in the GXXG motif of each KH domains, we showed that all four KH domains of CRD-BP are important for their physical association with MITF RNA. We mapped the CRD-BP-binding site in the 3’-UTR of MITF RNA from nts 1330–1740 and showed that the 49-nt fragment 1621–1669 is the minimal size MITF RNA for binding. Upon deletion of nts 1621–1669 within the nts1550-1740 of MITF RNA, there was a 3-fold increase in dissociation constant Kd, which further confirms the critical role sequences within nts 1621–1669 in binding to CRD-BP. Amongst the eight antisense oligonucleotides designed against MITF RNA 1550–1740, we found MHO-1 and MHO-7 as potent inhibitors of the CRD-BP-MITF RNA interaction. Using RNase protection and fluorescence polarization assays, we showed that both MHO-1 and MHO-7 have affinity for the MITF RNA, suggesting that both antisense oligonucleotides inhibited CRD-BP-MITF RNA interaction by directly binding to MITF RNA. The new molecular insights provided in this study have important implications for understanding the oncogenic function of CRD-BP and development of specific inhibitors against CRD-BP-MITF RNA interaction.

## Introduction

Coding region determinant-binding protein (CRD-BP; mouse), also known as IMP1 (human), belongs to a highly conserved family of RNA-binding proteins called VICKZ; all which possess two N-terminal RNA-recognition motifs (RRMs) followed by four C-terminal KH [hnRNP (heterogeneous nuclear ribonucleoprotein) K-homology] domains [1]. CRD-BP/IMP1 in human cancers has been studied extensively. CRD-BP is over-expressed in various human cancers, which include cancers of the breast, colon, brain, lung, testicular, skin, ovarian, and chorion [1]. A recent study demonstrated the existence of an N-terminal deleted CRD-BP isoform termed ΔN-CRD-BP. By employing an antibody against the C-terminal domain of CRD-BP, this isoform has shown to be ubiquitously expressed in normal adult tissues and is the major form elevated in breast tumours [2]. Two animal studies have demonstrate the oncogenic role of CRD-BP. Transgenic mice carrying targeted expression of CRD-BP develop mammary tumours [3] and overexpression of CRD-BP promotes xenograft tumour growth and dissemination into the blood in colorectal cancer cell xenografts [4].

The exact oncogenic mechanism whereby CRD-BP promotes tumour growth and metastasis is still unclear, but cumulative evidence suggests its ability to physically interact and stabilize oncogenic mRNAs is one important criterion. CRD-BP was first discovered due to its ability to physically interact with a specific coding region of c-*myc* mRNA, and its ability to influence c-*myc* mRNA stability was demonstrated in cell-free and cell line models [5–9]. Many studies have now confirmed the function of CRD-BP in binding to and shielding targeted mRNAs from degradation. For instance, CRD-BP binds with high affinity to the 3’-untranslated region (UTR) of CD44 mRNA to stabilize it, leading to cell adhesion, cytoplasmic spreading, and invadopodia formation [10]. CRD-BP binds to the coding region of βTrCP1 mRNA, and overexpression of CRD-BP led to the stabilization of βTrCP1 mRNA and elevation of βTrCP1 expression levels in colorectal cancer cells [8]. Similarly, CRD-BP has been shown to bind to and stabilize GLI1 mRNA leading to elevated GLI1 protein and proliferation of colorectal cancer cells [11]. CRD-BP has been shown to bind to the coding region and 3’ UTR of K-Ras mRNA and its overexpression led to increased K-Ras expression and colon cancer cell proliferation [12]. CRD-BP has high affinity for a coding region of the MDR1 mRNA [13] and sensitization of drug-resistant cells to chemotherapeutic drugs was observed upon CRD-BP-mediated MDR1 expression [14].

The 3’ UTR of microphthalmia-associated transcription factor (MITF) mRNA is also a binding site for CRD-BP and this interaction has been shown to be critical for protecting MITF transcript from degradation by miR-340, a mechanism believed to be important in melanocytes and melanoma [15]. MITF is a transcription factor, which normally controls the transcription of enzymes involved in pigmentation [16]. Amplification of the MITF oncogene in melanomas has been shown to induce the cell cycle and cell proliferation [17–19], as well as inhibit apoptosis [20]. Therefore, like CRD-BP, MITF represents a good target for anti-cancer therapy particularly in the treatment of melanoma.

In this study, we set out to understand the molecular interaction between CRD-BP and MITF RNA. We further mapped the 3’ UTR region of MITF mRNA previously shown to be a high affinity site for CRD-BP. Using CRD-BP KH variants with point mutation in the GXXG motif, we determined the KH domains critical for binding to MITF RNA. We designed and assessed eight specific antisense oligonucleotides against MITF RNA and found two, which are potent inhibitors of CRD-BP-MITF RNA interaction.

## Materials and methods

### Oligonucleotides and primers

Table 1 shows sequences of antisense oligonucleotides (MHO-1 to MHO-8) used in this study. Table 2 shows the sequences of primers used to amplify the DNA template for synthesizing all the MITF RNA fragments used in RNA mapping studies. All oligonucleotides and primers were synthesized by Integrated DNA Technologies (IDT) Inc. (Coralville, Iowa).

**Table 1:** Sequences of antisense oligonucleotides used in the competitive EMSA against MITF RNA.

Name	Sequences (5’ to 3’)
MHO-1	TAAAAATCTCAAATCAAGTTTC
MHO-2	TTTCTCTGACGCAAAATCCTCT
MHO-3	TTTCCAAGGAAGTCACAGGCAT
MHO-4	TTGCTCAGAAAGCTAAAGTCTG
MHO-5	TTCAATTAAACTTTACATTTGA
MHO-6	ATACTGCACACTTATTTGTACA
MHO-7	ATTCAGTTCACAGATACTGCAC
MHO-8	CCTGAATAAAAATGGACAGACA

**Table 2:** Sequences of primers used for amplifying MITF DNA fragments.

Name	Sequences (5’ to 3’)
MITF 1330–1550	Forward: GGATCCTAATACGACTCACTATAGGACACTTGTTAGCGAATCC; Reverse: TACTTTGGAGGCATTTGT
MITF 1550–1740	Forward: GGATCCTAATACGACTCACTATAGGATTGTACAAATAAGTGTG; Reverse: TGGTTGCTTTACATTCTT
MITF 1550–1709	Forward: As above for MITF 1550–1740; Reverse: ACA TTTGATTTCCAAGGA
MITF 1550–1669	Forward: As above for MITF 1550–1740; Reverse: AATCAAGTTTCCCCTGAA
MITF 1550–1619	Forward: As above for MITF 1550–1740; Reverse: TCCTCTTGCTCAGAAAGC
MITF 1550–1589	Forward: As above for MITF 1550–1740; Reverse: TGAATTCAGTTCACAGAT
MITF 1581–1740	Forward: GGATCCTAATACGACTCACTATAGGCTGAATTCACCACAGACT; Reverse: As above for MITF 1550–1740
MITF 1621–1740	Forward: GGATCCTAATACGACTCACTATAGGTTTGCGTCAGAGAAATGT; Reverse: As above for MITF 1550–1740
MITF 1701–1740	Forward: GGATCCTAATACGACTCACTATAGGATCAAATGTAAAGTTTAA; Reverse: As above for MITF 1550–1740

T7 RNA promoter sequences are underlined

### PCR-mediated deletion mutagenesis

To generate DNA fragment MITF 1550–1740 (Δ1621–1669), that is MITF nts 1550–1740 with deleted region from 1621–1669, we performed PCR-mediated deletion mutagenesis in 2 stages. High fidelity Taq polymerase and 10 μM of primers were used in all PCR reactions. In stage 1, two separate PCR reactions were performed to generate fragments 1550–1620 and 1670–1740 with flanking sequences. To generate fragment 1550–1620 with flanking sequences, 1550–1567 primer (5’-GGA TCC TAA TAC GAC TCA TAG GAT TGT ACA AAT AAG TGT G-3’) and 1620_reverse primer (5’-GGC ATA AAA ATC TCA ATC CTC TTG CTC AGA-3’) were used. To generate fragment 1620–1740 with flanking sequences, 1670_forward primer (5’-TCT GAG CAA GAG GAT TGA GAT TGA GAT TTT TAT GCC-3’) and 1723–1740 reverse primer (5’-TGG TTG CTT TAC ATT CTT-3’) were used. The PCR conditions used for both reactions were: 98°C for 30 sec followed by 30 cycles of 98°C 15 sec for denaturation, 42°C 30 sec for annealing and 72°C 45 sec for extension, and finally 72°C for 10 min. In stage 2, 1/100 dilutions of both of the above PCR-generated fragments 1550–1620 and 1670–1740 with flanking sequences were used as templates in a PCR reaction containing 1550–1567 forward and 1723–1740 reverse primers. The PCR conditions used for stage 2 were: one soft cycle of 98°C 45 sec, 42°C 30 sec, 72°C 1 min followed by 30 cycles of 98°C 15 sec, 50°C 30 sec, 72°C 1 min, and finally 72°C for 10 min. Upon visualization of PCR products on agarose gel, the faster migrating band which contained MITF 1550–1740 (Δ1621–1669) was gel sliced, purified and further amplified using 1550–1567 forward and 1723–1740 reverse primers. The PCR-amplified MITF 1550–1740 (Δ1621–1669) DNA fragment was then verified by DNA sequencing. The DNA fragment contains T7 promoter sequence at its 5’end was then used for *in-vitro* transcription as described below.

### Generation and purification of recombinant CRD-BP and its variants

The plasmid pET28b(+)-CRD-BP which contains the mouse CRD-BP cDNA was used to generate recombinant WT CRD-BP. The generation of various KH point mutation variants was accomplished using PCR-based site-directed mutagenesis method and has been previously described [21]. Recombinant CRD-BP was purified from *Escherichia coli* BL21 (DE3) using a 1 mL bed volume of nickel-NTA (QIAGEN) column under denaturing conditions. Proteins eluted from the column at pH 5.4 were subjected to three steps of dialysis. The first step was for 24 hours in pH 7.4 buffer containing 200 mM NaCl, 20 mM Tris-HCl, 1 mM reduced glutathione, 0.1 mM oxidized glutathione, 10% (v/v) glycerol, 2 M urea, and 0.01% (v/v) Triton X-100. Proteins were then dialyzed twice, each for 2 hours in the same buffer as above, but without urea and the glutathiones. Following dialysis, samples were spun at 13,200 rpm for 30 min to remove any precipitated proteins. The purified protein solutions were then quantified using Quick Start Bradford 1 x Dye Reagent (Bio-Rad, Mississauga, Ontario) or BCA Protein Assay Kit (ThermoFisher, Ottawa, Ontario), and analyzed for purity using Coomassie brilliant blue-stained 12% SDS-PAGE. The purified proteins were also confirmed by Western analysis using specific antibody against CRD-BP [21].

### Generation of DNA templates and radiolabeled *in-vitro* transcription

The human MITF (Clone ID 6066096; accession # BC065243) and β–TrCP1 (Clone ID 4419029; accession # BC026213) cDNAs purchased from Thermo Scientific Open Biosystems (Lafayette, CO) were used as templates for the generation of PCR-amplified DNA fragments. The different sets of forward and reverse primers used to amplify the different regions of MITF cDNA are shown in Table 2. PCR amplified DNA templates were used directly for *in-vitro* transcription by T7 RNA polymerase. One μg of DNA template was incubated for 1 h at 37°C in a 20-μl reaction containing 1 x transcription buffer (Promega, Madison, Wisconsin), 10 mM dithiothreitol, 1 unit RNasin (Promega), 0.5 mM ATP, 0.5 mM CTP, 0.5 mM GTP, 12.5 μM UTP, 1.5 units T7 RNA polymerase (Promega, Madison, WI), and 40 μCi [α-^32^P] UTP (3000 Ci/mmol). Following incubation, 3 units of RNase-free DNase I (Promega) were added, and the reaction was further incubated for 10 min at 37°C. Upon addition of 10 μl Stopping dye (9 M urea, 0.01% bromophenol blue, 0.01% xylene cyanole FF, 0.01% phenol), the entire sample was electrophoresed on a 8% polyacrylamide/7M urea gel. Bands containing internally-radiolabeled RNA and determined to be the correct size, were excised, and eluted with elution buffer (10 mM Tris-HCl pH 7.5, 0.1M NaCl, 1 mM EDTA, 0.01% SDS) at 45^°^C for 6 hours. The purified, radiolabeled RNA was then phenol/chloroform extracted followed by ethanol precipitation. Specific activity of the RNA was then determined by scintillation counting.

### Generation of unlabeled RNA

For the synthesis of unlabeled MITF RNA nts 1550–1740 and GLI1 RNA nts 346–382 for use in competitive electrophoretic mobility shift assay (EMSA), the following reactions and procedure were taken. Each *in-vitro* transcription reaction contained 5 μg of gel purified DNA template, 10 μL 10 x T7 buffer (400 mM Tris-HCl pH 7.6, 240 mM MgCl_2_, 20 mM spermidine, 0.1% Triton X-100), 5 μL each of 100 mM ATP, CTP, GTP, and UTP, 40 units RNasin, 5 units of T7 RNA polymerase to a total volume of 100 μL. The reaction was incubated at 37°C overnight followed by treatment with DNase I to remove the DNA template. Phenol-chloroform extraction was performed to remove protein impurities followed by passing through G-50 spin column to remove unincorporated nucleotides. RNA was ethanol precipitated and re-suspended in DEPC-treated water. Purity of RNA was checked by visualization on 2.5% agarose gel, and their concentration was determined using spectrophotometer.

### Electrophoretic mobility shift assay

The electrophoretic mobility shift assay (EMSA) was conducted in essentially the same manner as previously described [21,22]. EMSA competition assays involved the pre-incubation between competitor molecules (oligonucleotides or RNA) and 300 nM CRD-BP for 10 minutes at 35°C. Following the pre-incubation, 13 nM [^32^P]-labelled MITF RNA nts 1550–1740 was added to the reaction. This was followed by the standard EMSA protocol. The molar excess concentrations of antisense oligonucleotides MHO-1 to MHO-8, DD7 or GLI1 RNA nts 346–382 over the [^32^P]-labelled MITF RNA are shown in the competitive EMSA studies.

### RNase protection assay

DNA-RNA complexes were first generated by adding 20,000 cpm of ^32^P-labelled MITF 1550–1740 RNA probe to separate tubes containing 2 μg of DNA oligonucleotides (MHO-1 or MHO-7) or 2 μg yeast tRNA as control. The nucleic acid samples were precipitated and resuspended in 10 μl of Hybridization Buffer III (Ambion RPA III kit) followed by denaturation at 95°C for 3 min, and hybridization overnight at 42°C. Three 150 μl RNase H solutions containing 7.5 U of RNase H (New England Biolabs) in 1 X RNase H buffer were prepared and mixed with each hybridization reaction and incubated at 37°C for 1 hour, followed by heat inactivation at 65°C for 20 min. Digested RNA was then precipitated at -20°C for 1 hour after adding 225 μl of RNase Inactivation/precipitation III solution (Ambion RPA III kit), followed by 30 min centrifugation at 10,000 x g. RNA pellets were dried and suspended in 10 μl of Gel Loading Buffer II (RPA III kit) and fully denatured by heating at 95°C for 3 min. RNA samples were then resolved by gel electrophoresis on an 8% denaturing (7 M urea) polyacrylamide gel and exposed overnight to phosphor screen before visualization using the Cyclone Plus phosphorimager system (PerkinElmer).

### Fluorescence polarization assay

The following fluorescein-labeled oligonucleotides were used: MHO-1_FL (5’-TAA AAA TCT CAA ATC AAG TTT C/36-FAM/-3’); MHO-7_FL (5’-ATT CAG TTC ACA GAT ACT GCA C/36-FAM/-3’); MHO-6_FL (5’-ATA CTG CAC ACT TAT TTG TAC A/36-FAM/-3’). As a positive control, we used 39-nt fluorescent-labeled CD44 (FL-CD44) RNA (5’- AAA UUA GGG CCC AAU UAA UAA UCA GCA AGA AUU UGA UCG /36-FAM/ -3’) which is known to bind to CRD-BP. All fluorescein-labeled nucleic acids were synthesized by IDT Inc. For assessment of binding to CRD-BP, the following reaction mixture was used: 4 μl of binding buffer (10 mM Tris-HCl pH7.4, 2.5 mM EDTA, 5% glycerol, 0.002% Triton-X 100), 7 μl recombinant CRD-BP (0–1000 nM), 5 μl fluorescein-labeled nucleic acids (10 nM), and 3 μl sterile water. The mixtures were added to 384 Microfluor Microtiter plate (Thermo Electron) and incubated at 37°C for 30 min prior to plate reading. For assessment of binding to MITF RNA 1550–1740, the following mixture was used: 4 μl Hybridization Buffer III (Ambion RPA III kit), 7 μl MITF RNA 1550–1740 (0–1000 nM), and 8 μl fluorescein-labeled nucleic acids (10 nM). The mixtures were incubated at 42°C for 4 hours to allow nucleic acid binding. Plates were read at excitation wavelength 485 nm and at emission wavelength 528 nm using Gen5 software in Synergy 2 (Bio-Tek, Vermont).

## Results

### Mapping the MITF RNA regions that bind CRD-BP

**Fig 1:**
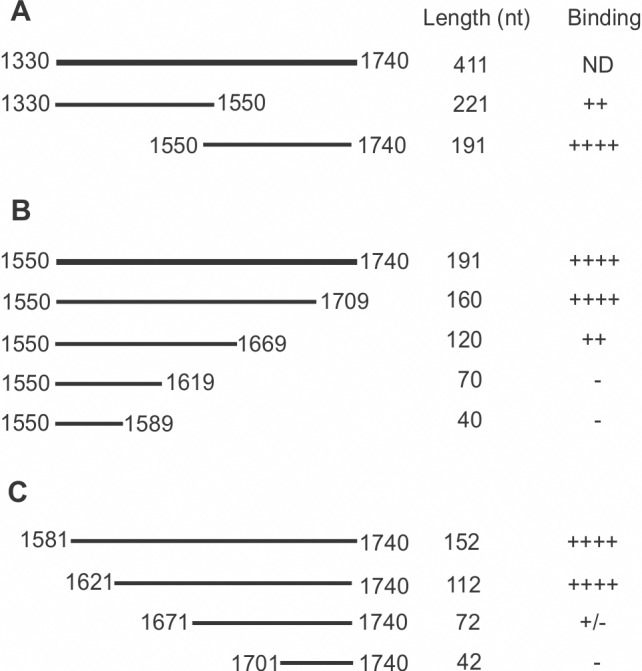
Summary of MITF RNA fragments and their relative binding affinity for CRD-BP. (A) Two fragments of DNA corresponding to MITF cDNA nts 1330–1550 and 1550–1740 were PCR-amplified with the appropriate primers shown in Table 2. (B) Four fragments of 3’ end truncated DNA corresponding to MITF cDNA nts 1550–1740 were generated as in (a) using the appropriate primers listed in Table 2. (C) Four fragments of 5’ end truncated DNA corresponding to MITF cDNA nts 1581–1740 were generated using the appropriate primers shown in Table 2. ^32^P] RNA was prepared from each of the above DNA template and analyzed by electrophoretic mobility shift assay (EMSA) for CRD-BP binding shown in Fig 2. (++++), (+++), (++), and (+) indicate ~90%, ~70%, ~40% and ~10% binding respectively. (+/-) and (-) indicate weak and no protein-RNA complex formation respectively.

**Fig 2:**
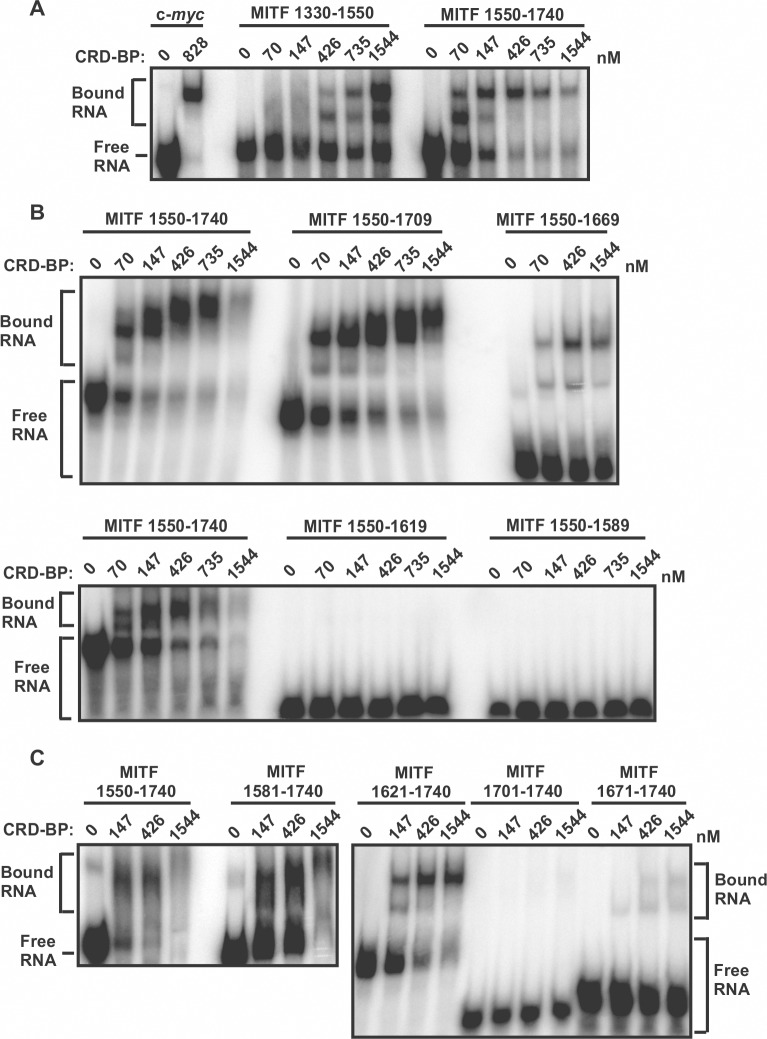
Mapping the CRD-BP binding site on MITF RNA using electrophoretic mobility shift assay. (A) Two fragments (nts 1330–1550 and 1550–1740) of [^32^P] MITF RNA (13 nM) covering nts 1330–1740 of MITF cDNA were incubated with various concentrations of recombinant CRD-BP as shown. [^32^P] c-*myc* RNA nts 1705–1886 was used as a positive control. (B) Four 3’ end truncated fragments (nts 1550–1709, 1550–1609, 1550–1619, and 1550–1589) of [^32^P] MITF RNA covering nts 1550–1740 of MITF cDNA were incubated with various concentrations of recombinant CRD-BP and analysed using EMSA as shown. The [^32^P] MITF RNA nts 1550–1740 was used for comparison. (C) As above, four 5’ end truncated [^32^P] MITF RNAs (nts 1581–1740, 1621–1740, 1671–1740, and 1701–1740) were assessed for their ability to bind CRD-BP. Samples were electrophoresed in a 4% native polyacrylamide followed by autoradiography. In each gel, free RNA and CRD-BP-bound RNA are indicated.

Using the method of UV cross-linking coupled with immuno-precipitation, it was previously shown that CRD-BP has affinity for MITF mRNA at the 3’ UTR spanning nts 1261–1822 [15]. To begin mapping the smallest region within the 3’ UTR of MITF that still binds CRD-BP, we first generated two RNA fragments corresponding to nts 1330–1740 of MITF mRNA. The two RNA fragments correspond to MITF mRNA at nts 1330–1550 and 1550–1740 (Fig 1A). The results of the electrophoretic mobility shift assay (EMSA) in assessing the ability of five concentrations of CRD-BP in binding these two radiolabeled MITF RNA fragments are shown in Fig 2A. As a positive control, we used the ^32^P-labeled c-*myc* CRD RNA nts 1705–1886 which has high affinity for CRD-BP [21]. As shown in Fig 2A, addition of CRD-BP resulted in protein-c-*myc* RNA complex. Amongst the two MITF RNA fragments tested, we found MITF nts 1550–1740 to have higher affinity for CRD-BP with clear binding occurring at 70 nM CRD-BP (Fig 2A). This was in contrast to the MITF RNA nts 1330–1550, which showed binding only when 426 nM CRD-BP was added (Fig 2A).

**Fig 3:**
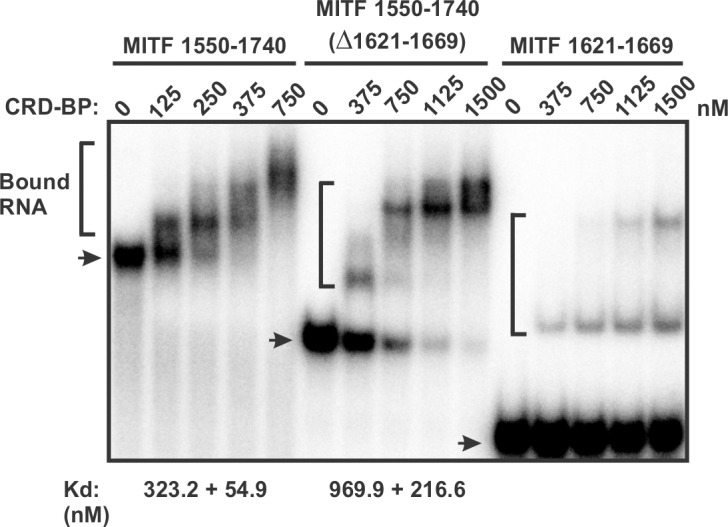
Assessing the role of nucleotides 1621–1669 of MITF RNA in binding CRD-BP. Electrophoretic mobility gel-shift assay on the binding of purified recombinant WT CRD-BP to MITF RNA 1550–1740, MITF RNA 1550–1740 (Δ1621–1669) and MITF RNA 1621–1669. Various concentrations of CRD-BP, as shown, were incubated with 40 nM [^32^P] MITF RNA. Arrows indicate the free RNA and the brackets indicate CRD-BP-bound RNA. Dissociation constant (Kd) for CRD-BP to bind MITF RNA 1550–1740 and MITF RNA 1550–1740 (Δ1621–1669) are shown at the bottom of the image. The Kd for binding to MITF RNA 1621–1669 could not be determined.

Based on the finding that MITF RNA nts 1550–1740 has higher affinity for CRD-BP, we next further determined the smallest RNA fragment within this region that still binds CRD-BP. To do this, we generated four RNA fragments truncated from the 3’ end (Fig 1B) and four RNA fragments truncated from the 5’ end of nts 1550–1740 (Fig 1C). The results of the EMSA in assessing the RNA fragment’s affinity for CRD-BP are shown in Fig 2B and 2C. Amongst the 3’ end truncated fragments, MITF RNA fragments nts 1550–1709 and nts 1550–1669 demonstrated the ability to bind CRD-BP (Fig 2B). The two smaller MITF RNA fragments nts 1550–1619 and nts 1550–1589 were incapable of binding CRD-BP (Fig 2B). Amongst the 5’ end truncated fragments, we found two, nts 1581–1740 and 1621–1740, that had strong affinity for CRD-BP (Fig 2C). MITF RNA nts 1671–1740 had weak affinity for CRD-BP while nts 1701–1740 clearly could not bind CRD-BP (Fig 2C). Based on the combined experimental data from 3’ end and 5’ end truncated MITF RNA fragments nts 1550–1740 (Fig 2), we propose that the RNA region corresponding to nts 1621–1669 (49 nts size) might still bind to CRD-BP and represent an important sequence element for binding to CRD-BP. To test this hypothesis, we generated the MITF RNA 1621–1669 and directly perform EMSA to determine if it still binds to CRD-BP. Indeed, our results show that CRD-BP still has affinity for the 49-nt RNA even at 375 nM (right side of the image in Fig 3). Hence, we concluded the smallest region that was experimentally determined to bind CRD-BP is the 49-nt fragment spanning nts 1621–1669. To further determine the significance of nts 1621–1669, we compare the CRD-BP-binding profile of MITF RNA 1550–1740 with MITF RNA 1550–1740 (Δ1621–1669). As shown in Fig 3, MITF RNA 1550–1740 with the deleted 1621–1669 had 3-fold lower affinity (compare Kd of 969.9 ± 216.6 nM to 323.2 ± 54.9 nM) for CRD-BP than the full-length 1550–1740. Please refer to the binding curves in S1 Fig for obtaining the Kd values and the raw data sets in S1 Table for generating S1 Fig. This result is consistent with the notion that nts 1621–1669 is an important element at the 3’UTR of MITF RNA in binding to CRD-BP.

### Assessing the role of the KH domains of CRD-BP in binding MITF RNA

**Fig 4:**
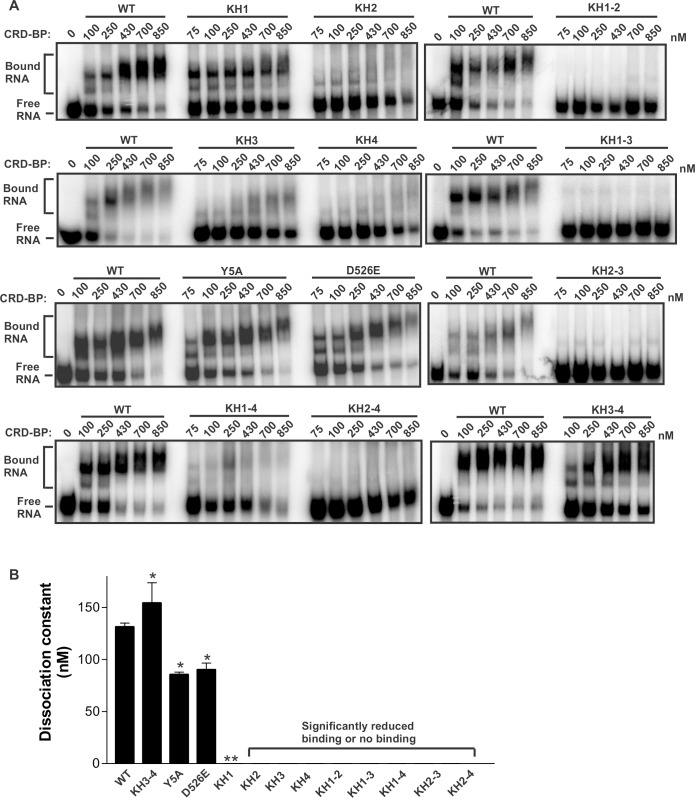
Binding profiles of CRD-BP and its point mutation variants on MITF RNA. (A) Electrophoretic mobility gel-shift assay on the binding of purified recombinant WT CRD-BP and its point mutation variants to [^32^P] MITF RNA nts 1550–1740 (191 nts). Various concentrations of proteins, as indicated, were incubated with 40 nM of the radiolabeled MITF RNA. Samples within each panel indicate results from the same gel generated from the same experiment. Data shown are representatives from at least three experiments using at least two separately prepared recombinant proteins. The positions of protein-RNA complexes (Bound) and free RNA are indicated. (B) A summary of dissociation constants (K_d_) of the WT CRD-BP and its variants. The K_d_ values were taken from saturation binding curves (n = 4). The single asterisk indicates that the p-value is less than 0.05 based on Student’s t-test in comparing to the K_d_ of WT CRD-BP. The double asterisks indicate that there was significant reduction and hence the K_d_ could not be determined.

To date, most deletion studies on paralogs of CRD-BP have shown that the two RRM domains are not important for RNA binding [23–25]. Hence, our study focused predominantly on the four KH domains of CRD-BP. Using site-directed mutagenesis to mutate the first glycine to an aspartate in the GXXG motif of KH domains, we recently showed that at least two KH domains of CRD-BP participated in binding c-*myc* and CD44 RNAs [21]. Here, we were interested in determining whether CRD-BP has similar requirements for binding MITF RNA. We used the previously described single point-mutation KH variants (KH1, KH2, KH3, and KH4) and double point-mutation KH variants (KH1-2, KH1-3, KH1-4, KH2-3, KH2-4, and KH3-4) [21]. As additional controls, we used Y5A variant, which has point mutation in the RRM1 domain and D526E variant with point mutation in the variable loop located between β_2_ and β_3_ in KH4 domain [21]. The representative results of EMSA in assessing these variants for binding to [^32^P]-labelled MITF RNA nts 1550–1740 are shown in Fig 4A, and the data summarized from three biological replicates is shown in Fig 4B. Please see S2 Fig for the binding curves in obtaining the Kd values. Both control variants, Y5A (85 ± 2 nM) and D526E (91 ± 6 nM), exhibited dissociation constant, K_d_, which is lower than that of the WT CRD-BP (132 ± 3 nM), suggesting that the control variants have slightly higher affinity for MITF RNA. Surprisingly, the KH single variants, KH2, KH3 and KH4, had abolished ability to bind MITF RNA (Fig 4). The KH1 variant could bind MITF RNA, however its binding profile does not follow the usual saturation binding curve as exhibited by the WT CRD-BP, and hence its K_d_ value cannot be determined (Fig 4). All the di-domain variants, with the exception of KH3-4 variant, had abolished ability to bind MITF RNA. The KH3-4 variant had slightly lower affinity for MITF RNA as compared to the WT CRD-BP, with a K_d_ of 155 ± 19 nM. It is important to point out that although proteins were purified to 95% homogeneity, there was non-specific aggregation of RNA-protein complex observed in wells of the gel when higher concentrations of some proteins were used. However, this has no bearing on our qualitative analysis of data in Fig 2 nor it changed the conclusion of the results shown in Fig 4.

### Assessing a panel of antisense oligonucleotides for ability to inhibit CRD-BP-MITF RNA interaction

**Fig 5:**
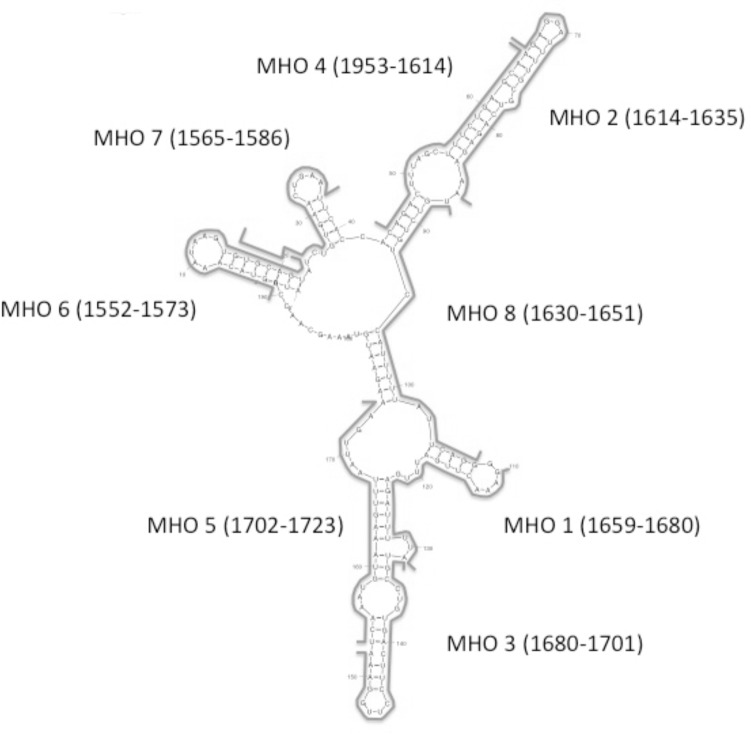
Predicted RNA secondary structure of MITF RNA and target sites of antisense oligonucleotides. The predicted secondary structure of MITF RNA (nts 1550–1740) was generated using the MFOLD program [27]. As shown, the solid lines indicate complimentary regions where each of the 8 MHO antisense oligonucleotides hybridizes to. The number within the bracket indicates the specific nucleotide sequences where the MHO antisense oligonucleotides target.

**Fig 6:**
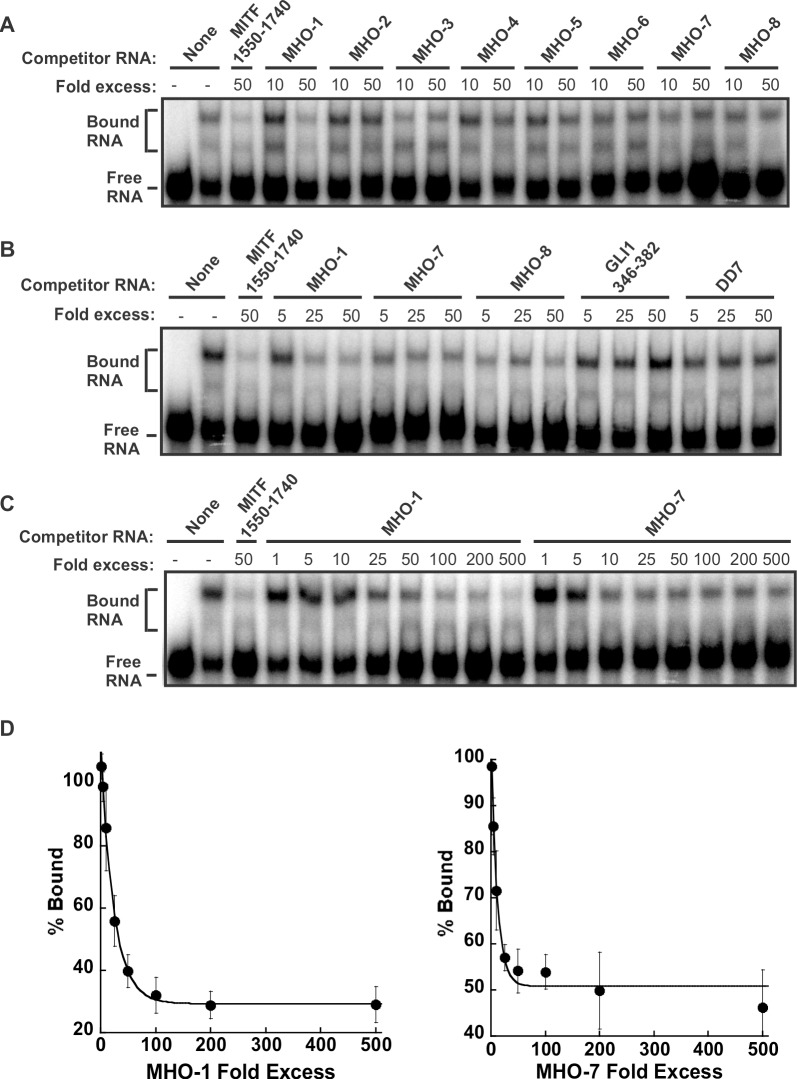
Inhibition of the CRD-BP-MITF RNA interaction by antisense oligonucleotides. (A) Purified recombinant CRD-BP (2000 nM) was incubated with [^32^P] MITF RNA nts 1550–1740 in the presence of antisense oligonucleotides MHO-1 to MHO-8 at 10- or 50-fold molar excess. As a positive control, 50-fold molar excess of unlabelled MITF RNA nts 1550–1740 (lane 3) was used. (B) As in (A), 5-, 25-, and 50-fold molar excess of antisense oligonucleotides MHO-1, MHO-7, MHO-8, GLI1 RNA nts 346–382 and DD7 oligonucleotide was competed with [^32^P] MITF RNA nts 1550–1740 for binding to CRD-BP. (C) A wider concentration range of antisense oligonucleotides MHO-1 and MHO-7 at 1- to 500-fold molar excess were used in the competitive EMSA against [^32^P] MITF RNA nts 1550–1740. Samples were electrophoresed in a 4% native polyacrylamide followed by autoradiography. In each gel, free RNA and CRD-BP-bound RNA are indicated. (D) The average percentage of bound complex, taken from (C) and two other separate experiments is expressed on the graph as shown.

We next investigated the possibility of using antisense oligonucleotides to inhibit the CRD-BP-MITF RNA interaction. We opted to use the DNA oligonucleotides instead of the RNA versions mainly because of their known stability and therefore consistency in results. We designed eight 22-nt antisense oligonucleotides, termed MHO-1 to MHO-8 (Table 1), which corresponded to the sequence of MITF RNA nts 1550–1740 (Fig 5) and assessed their ability to inhibit the binding of ^32^P-labeled MITF RNA to CRD-BP. The regions targeted by each of the antisense oligonucleotides are shown in Fig 5 and the results of the competitive EMSA are shown in Fig 6. At 50-fold molar excess, the positive control unlabeled MITF RNA nts 1550–1740 successfully inhibited CRD-BP-MITF interaction (Fig 6A). Amongst the eight antisense oligonucleotides, MHO-1, MHO-3, MHO-7 and MHO-8, appeared to be effective inhibitor at either 10- or 50-fold molar excess (Fig 6A). Subsequent studies showed that the effect of MHO-3 was less reproducible and therefore was not studied further. Using a wider concentration range of the oligonucleotides, the effectiveness of MHO-1, MHO-7 and MHO-8 as inhibitors of CRD-BP-MITF RNA interaction was confirmed (Fig 6B). Such inhibition is specific since both DD7 and GLI1 nts 346–382 oligonucleotides had no effect on CRD-BP-MITF RNA interaction even up to 50-fold molar excess (Fig 6B). DD7 is a specific inhibitor of CRD-BP-CD44 RNA interaction [22] while GLI1 nts 346–382 is a specific inhibitor of CRD-BP-GLI1 RNA interaction [26]. We further showed that MHO-1 is a more effective inhibitor because it could inhibit 50% bound MITF RNA at 25-fold molar excess while MHO-7 required about 50-fold molar excess to achieve similar level of inhibition (Fig 6C and 6D). Please see raw data sets in S2 Table for generating Fig 6D.

### Evidence that MHO-1 and MHO-7 bind to MITF RNA

**Fig 7:**
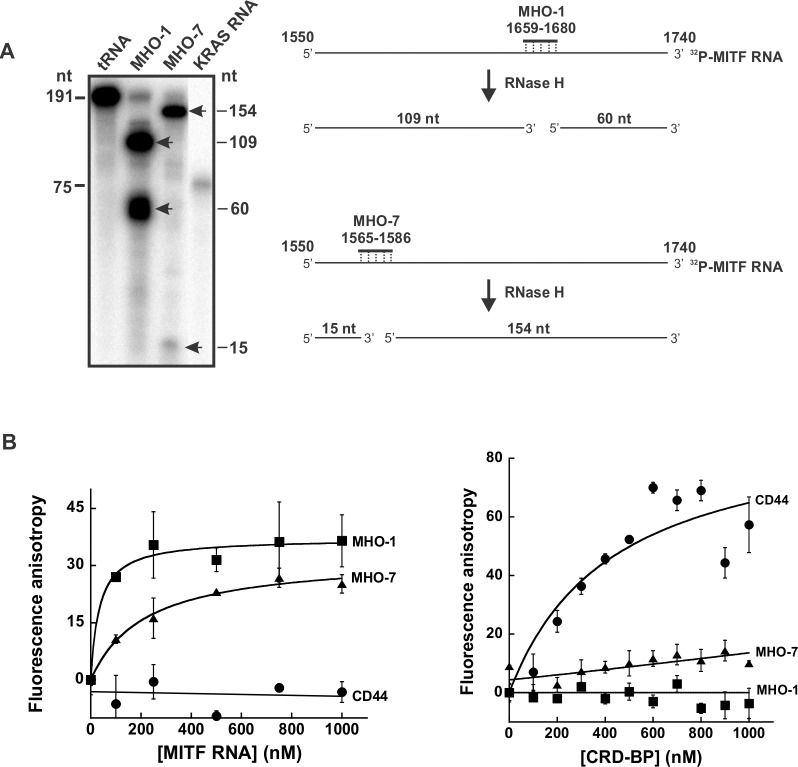
Assessing the binding of MHO-1 and MHO-7 to MITF RNA and CRD-BP. (A) RNase protection assay to assess the binding of MHO-1 and MHO-7 antisense oligonucleotides to MITF RNA 1550–1740. ^32^P-MITF RNA 1550–1740 was incubated with tRNA, MHO-1 or MHO-7 overnight at 42°C as described in Materials and Methods. The reactions were then subjected to RNase H treatment, precipitated, and ran on 8% denaturing polyacrylamide gel as shown. ^32^P-MITF RNA fragments which were not digested by RNase H are shown by arrows. The 75 nts size KRAS RNA on lane 4 was used as a marker. The schematic diagram on the right illustrates the action of RNase H and the expected RNA fragments generated upon hybridization of MHO-1 and MHO-7 to ^32^P-MITF RNA 1550–1740. (B) Fluorescence polarization assays to assess the binding of MHO-1 and MHO-7 to MITF RNA (left panel) and CRD-BP (right panel). Ten nM of fluorescein-labeled MHO-1 and MHO-7 AONs as well as fluorescein-labeled CD44 RNA were incubated with an increasing concentration of MITF RNA (0–1000 nM) (left panel) or CRD-BP (0–1000 nM) (right panel) as described in Materials and Methods. The error bars are S.E. and the results are representative of two separate experiments.

To help understand the mechanism whereby the antisense oligonucleotides MHO-1 and MHO-7 inhibit CRD-BP-MITF RNA interaction, we first performed RNase protection assay to determine if both antisense oligonucleotides can bind to MITF RNA 1550–1740 in which they were designed against (Fig 5). We used RNase H in our RNase protection assay because it degrades DNA-RNA hybrids. The diagrams on the right panel in Fig 7A depict the expected sized fragments generated by RNase H upon hybridization of MHO-1 and MHO-7 to the ^32^P-MITF RNA 1550–1740. The results from our RNase protection assay (left panel in Fig 7A) show that RNase H generated two RNA fragments of expected size when the 191 nt ^32^P-MITF RNA 1550–1740 was incubated with MHO-1 and MHO-7. These results demonstrated that both MHO-1 and MHO-7 are indeed capable of binding to the MITF RNA.

From our past experiences, EMSA is typically a more suitable method for studying CRD-BP-nucleic acid interaction if the nucleic acid is of larger size (e.g., see Figs 2 and 3). Hence, when the 22-nt antisense oligonucleotides MHO-1 and MHO-7 were evaluated for binding to CRD-BP in EMSA, no clear binding was observed (data not shown). This could be because MHO-1 and MHO-7 cannot bind to CRD-BP or simply because EMSA is not sensitive enough and hence unsuitable for studying CRD-BP-small oligonucleotide interaction. Therefore, we turned to a more sensitive fluorescence polarization method to detect such interaction. As shown on the left panel in Fig 7B (the raw data sets are in S3 Table), there was increasing fluorescence units when increasing amounts of MITF RNA 1550–1740 was incubated with fluorescein-labeled MHO-1 and MHO-7, indicating the binding of these AONs to MITF RNA. In contrast, the fluorescein-labeled 39-nt CD44 RNA used as a negative control had no effect. This result further confirms the conclusion drawn from the above RNase protection assay. The results on the left panel in Fig 7B (the raw data sets are in S3 Table) show that no increased in fluorescence units when increasing amount of CRD-BP were incubated with both fluorescein-labeled MHO-1 and MHO-7, suggesting that both AONs cannot bind to CRD-BP. On the other hand, the fluorescein-labeled 39-nt CD44 RNA used as a positive control [22] showed binding to CRD-BP. In summary, we conclude that both MHO-1 and MHO-7 are capable of binding to MITF RNA 1550–1740 and had no affinity to CRD-BP.

## Discussion

A recent study demonstrated CRD-BP as an important positive regulator of MITF expression and function in melanoma cell lines [15]. While miR-340 caused destabilization of MITF mRNA and decreased in MITF expression and transcriptional activity, overexpressed CRD-BP was able to reverse such miR-340-mediated effects [15]. Using FLAG immunoprecipitation of UV-cross-linked RNP complexes isolated from cells transfected with FLAG-CRD-BP, it was shown that CRD-BP has high affinity for the 3’ UTR of MITF mRNA spanning nts 1261–1822 [15]. This is consistent with the finding that 3’ UTR of MITF has binding sites for miR-340 and the results that CRD-BP can prevent miR-340-mediated degradation of MITF mRNA [15]. Based on these findings, we set out to understand the molecular interaction between CRD-BP and MITF RNA with the aim to further understand the oncogenic function of CRD-BP and to find new specific inhibitors against the onco-proteins.

By employing KH variants with a point mutation in the GXXG motif at each of the KH domains, we previously showed that, generally, the single point mutation in each of the four KH domains had no major impact on the ability of CRD-BP to bind to both c-*myc* and CD44 RNAs [21]. In this study, on the contrary, the single point mutation KH variants, KH2, KH3 and KH4, had completely abolished ability to bind MITF RNA (Fig 4 and Table 3), suggesting that the GXXG motif at the respective domains play critical role in associating CRD-BP with MITF RNA. The KH1 variant exhibited binding, however, its Kd cannot be determined due to the retardation in binding (Fig 4 and Table 3). All the KH variants with double mutations, with the exception of KH3-4 variants, showed complete abolition in binding MITF RNA. Similar findings were found with c-*myc* and CD44 RNAs (Table 3) [21], further supporting the notion that regions other than the GXXG motif within the KH3 and KH4 domains participate in binding RNAs. However, this does not hold true for all RNAs, as we have found complete inability of the KH3-4 variant to bind GLI1 [26] and KRas (Mackedenski et al., unpublished observation) RNAs (Table 3). Overall, our site-directed mutagenesis studies on the binding of KH variants on five RNA targets of CRD-BP, including MITF RNA, showed that indeed CRD-BP utilizes the different KH domains in binding distinct RNAs. This indicates that one hat does not fit all and suggests that any future CRD-BP inhibitor studies need to focus on the specific RNA target in question. Although our studies utilizes the *in vitro* EMSA, we consider this to be relevant since there is evidence that molecules that can break CRD-BP-RNA interaction *in vitro* also have the ability to decrease targeted mRNA expression in cells. This is exemplified in studies with CD44 [22], GLI1 [26], and KRas mRNAs (Mackedenski et al., unpublished observation).

**Table 3:** A summary of the dissociation constants (Kd) of WT CRD-BP in comparison with its KH variants for binding to several RNA targets.

CRD-BP variant	RNA targets				
	c-*myc*^1^	MITF	CD44^1^	GLI1^2^	KRas^3^
WT	398 ± 52.8	132 ± 3.36	149 ± 8.42	288 ± 23.5	178 ± 18.4
KH1	723 ± 75.1	NA^4^	211 ± 34.9	NA	NA
KH2	360 ± 70.4	NA	250 ± 26.7	NA	NA
KH3	321 ± 42.8	NA	219 ± 19.4	466 ± 136	NA
KH4	320 ± 59.1	NA	NA	202 ± 10.8	NA
KH1-2	NA	NA	NA	NA	NA
KH1-3	NA	NA	NA	NA	NA
KH1-4	NA	NA	NA	NA	NA
KH2-3	NA	NA	NA	NA	NA
KH2-4	NA	NA	NA	NA	NA
KH3-4	587 ± 42.9	155 ± 19.0	133 ± 11.0	NA	NA
Y5A	468 ± 45.7	85.0 ± 2.00	100 ± 16.4	384 ± 90.4	ND
D526E	402 ± 37.3	91.0 ± 6.00	90.7 ± 1.64	314 ± 67.1	ND
Q84A	491 ± 20.0	ND	ND	ND	ND
E445D	459 ± 42.2	ND	ND	ND	ND

NA indicates not applicable because significantly reduced or no binding occurred and hence the binding curve cannot be plotted. ND indicates not determined. Data are means ± S.E (n = 4).

^1^Adapted from reference 21

^2^adapted from reference 26

^3^adapted from Mackedenski et al., unpublished observation

^4^Binding but saturation binding curves could not be plotted.

The previous study showed that CRD-BP has affinity for the 562-nt of 3’ UTR of MITF 1261–1822 [15]. In this study, we mapped this MITF RNA to identify the smallest region that still binds CRD-BP. Our results demonstrated that the 49-nt MITF RNA 1621–1669 is the minimal region within nts 1550–1740 of 3’UTR of MITF RNA for binding CRD-BP (Fig 3). Our findings that deletion of 1621–1669 within the 1550–1740 nts led to 3-fold decrease in Kd further support the notion that sequences within 1621–1669 nts play an important role in binding CRD-BP (Fig 3). In our opinion, although the 49-nt sequence (1621–1669) is the minimal MITF fragment for binding to CRD-BP, it may not be indispensable in the control of MITF mRNA stability by CRD-BP. This is because CRD-BP also has affinity for nts 1330–1550 (Fig 2) which is parts of the 3’UTR and lies outside of the minimal sequence. In addition, the affinity of CRD-BP for the 49-nt minimal sequence is not high as compared to the larger RNA fragments such as nts 1621–1740 (Fig 2) and deletion of nts 1621–1669 did not abolish the affinity of nts 1550–1740 for CRD-BP (Fig 3). We also speculate that the minimal 49-nt sequence is not likely to involve in the miR-340-mediated MITF mRNA decay. This is because the two miR-340 binding sites (1444–1464 and 1848–1871) lie outside of the minimal 49-nt sequence (1621–1669) [15].

The mapping studies were important because it has allowed us to know which regions are critical for CRD-BP binding and hence the better design of antisense oligonucleotides (AONs) against MITF RNA (Fig 5), and subsequently the discovery of two potent AON inhibitors of CRD-BP-MITF RNA interaction, which we have termed MHO-1 and MHO-7 (Fig 6). Knowledge of the smallest MITF RNA fragment that binds CRD-BP also permits the development of fluorescent polarization method to conveniently study CRD-BP-MITF RNA interaction and to screen for small molecule inhibitors of the interaction. In this study, we further investigated the possible mechanism whereby MHO-1 and MHO-7 inhibit CRD-BP-MITF RNA interaction. Our results using RNase protection and fluorescence polarization assays (Fig 7) demonstrated that both AONs are capable of binding to the MITF RNA. In contrast, using the fluorescence polarization method (Fig 7B) we found no evidence that both AONs could bind to CRD-BP. Therefore, we propose that both MHO-1 and MHO-7 inhibit CRD-BP-MITF RNA interaction by directly binding to MITF RNA.

In conclusion, we have characterized the interaction between CRD-BP and MITF RNA nts 1550–1740 and mapped the MITF RNA region down to 49 nts in size, which still has affinity for CRD-BP. Our deletion studies further confirmed the importance of nts 1621–1669 within the 3’UTR of MITF RNA in binding to CRD-BP. Furthermore, we revealed two antisense oligonucleotides that can block the CRD-BP-MITF RNA interaction *in vitro* by binding to the MITF RNA. In addition to examining RNAs, we also show that all four KH domains are required by CRD-BP to bind MITF RNA. Taken together, this study has provided new insights into the CRD-BP-MITF RNA interaction. Such information will inevitably aid in future development of inhibitors of CRD-BP function.

## Supporting information

S1 Fig CRD-BP binding curves in obtaining Kd values for Fig 3. (TIF)

S2 Fig Binding curves for obtaining Kd values for Fig 4B. (TIF)

S1 Table Raw data sets for generating the binding curves in S1 Fig. (DOCX)

S2 Table Raw data sets for generating Fig 6D. (DOCX)

S3 Table Raw data sets for generating Fig 7B. (XLSX)
