# Supplementary material for: Characterizing the Coding Region Determinant-Binding Protein (CRD-BP)-Microphthalmia-associated Transcription Factor (MITF) mRNA interaction
Source: PLoS One. 2017 Feb 9;12(2):e0171196. doi: 10.1371/journal.pone.0171196 (PMC5300761; doi:10.1371/journal.pone.0171196)
Supplement: S2 Table — (DOCX) [file pone.0171196.s004.docx]

**S2 Table. Raw data set for generating Fig 6D**
